# Under the Mind's Hood: What We Have Learned by Watching the Brain at Work

**DOI:** 10.1523/JNEUROSCI.0742-19.2019

**Published:** 2020-01-02

**Authors:** Anna Christina Nobre, Freek van Ede

**Affiliations:** ^1^Oxford Centre for Human Brain Activity, Wellcome Centre for Integrative Neuroimaging, Department of Psychiatry, University of Oxford, Oxford OX3 7JX, United Kingdom, and; ^2^Department of Experimental Psychology, University of Oxford, Oxford OX2 6GG, United Kingdom

**Keywords:** Human brain imaging, Human neurophysiology, Historical overview, Selective attention, Functional Magnetic Resonance Imaging (fMRI), Electroencephalograhy (EEG)

## Abstract

Imagine you were asked to investigate the workings of an engine, but to do so without ever opening the hood. Now imagine the engine fueled the human mind. This is the challenge faced by cognitive neuroscientists worldwide aiming to understand the neural bases of our psychological functions. Luckily, human ingenuity comes to the rescue. Around the same time as the Society for Neuroscience was being established in the 1960s, the first tools for measuring the human brain at work were becoming available. Noninvasive human brain imaging and neurophysiology have continued developing at a relentless pace ever since. In this 50 year anniversary, we reflect on how these methods have been changing our understanding of how brain supports mind.

## Abstract (precis)

Imagine you were asked to investigate the workings of an engine, but to do so without ever opening the hood. Now imagine the engine fueled the human mind. This is the challenge faced by cognitive neuroscientists worldwide aiming to understand the neural bases of our psychological functions. Luckily, human ingenuity comes to the rescue.

## Introduction

**Figure 1.**
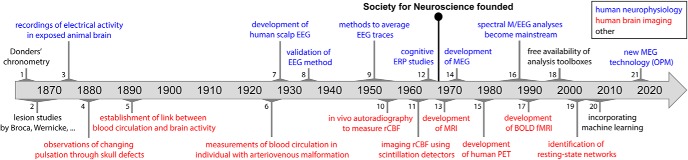
Timeline of key methodological developments in the history of going under the mind's hood. Timings are approximate, appreciating that many of the listed developments spanned several years, or involved relatively gradual transitions rather than abrupt events with a clearly marked onset and offset. Selected representative references for the listed events (ordered by their associated number in the schematic) are as follows: (1) Donders, 1869. (2) Broca, 1861; Wernicke, 1874. (3) Caton, 1875. (4) Mosso, 1881. (5) Roy and Sherrington, 1890. (6) Fulton, 1928. (7) Berger, 1929. (8) Adrian and Matthews, 1934. (9) Davis, 1939; Dawson, 1954; Galambos and Sheatz, 1962. (10) Landau et al., 1955. (11) Ingvar and Risberg, 1965. (12) Walter et al., 1964; Sutton et al., 1965. (13) Bloch, 1946; Purcell et al., 1946; Lauterbur, 1973; Mansfield, 1977. (14) Cohen, 1972. (15) Frackowiak et al., 1980; Raichle et al., 1983. (16) Pfurtscheller and Aranibar, 1979; Pfurtscheller and Lopes da Silva, 1999; Tallon-Baudry and Bertrand, 1999. (17) Ogawa et al., 1990, 1992; Kwong et al., 1992; Bandettini et al., 1992. (18) Friston et al., 1994; Smith et al., 2004; Oostenveld et al., 2011. (19) Biswal et al., 1995; Raichle et al., 2001; Fox et al., 2005. (20) Haxby et al., 2001; Kamitani and Tong, 2005; Kriegeskorte et al., 2006. (21) Boto et al., 2018.

The human mind — what could hold more mystery and fascination? For millennia, humans have puzzled and pondered over its origins and workings; but only over the last 50 years or so, have scientists had the experimental tools to go under the hood to measure its organ at work. For a timeline of key methodological developments, see Figure 1.

The foundations for our understanding of how mental functions are organized in the human brain come from neuropsychological studies based on observations of behavioral impairments and dissociations following naturally occurring brain lesions (e.g., Broca, 1861; Harlow, 1868; Wernicke, 1874; Loeb, 1885). None should deny the importance of human lesion studies in identifying the functional elements of the human mind as well as their interrelations and causal reliance upon specialized brain areas (e.g., Scoville and Milner, 1957). Yet, the method's limitations for investigating the workings of the brain are clear.

**Figure 2.**
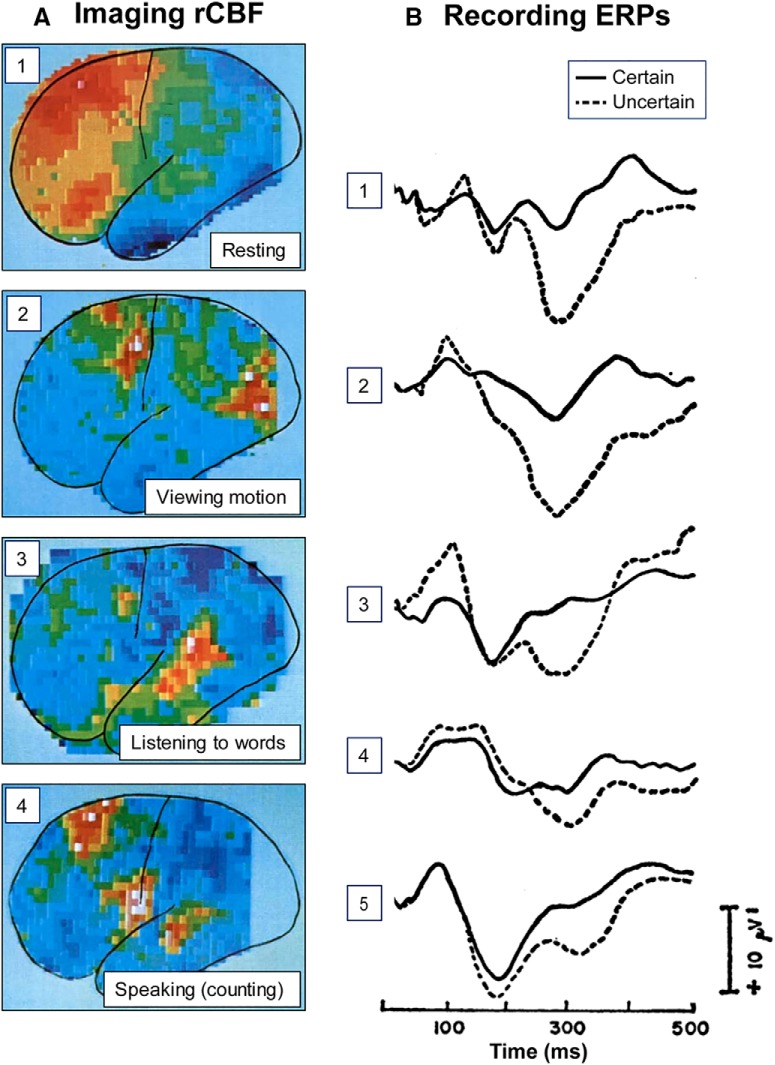
The first glimpses of cognition at work. Early results from (***A***) imaging and (***B***) recording human brain activity. ***A***, Patterns of cerebral blood flow measured using a scintillation detector placed next to a participant's head after injection of a radioactive isotope to detect its passage through the cortex. On the color scale: Green represents the mean flow rate. Shades of blue represent up to 20% decreases from mean. Shades from yellow to red represent up to 20% above mean flow rates. Images are maps from individual participants. (1) At rest, the brain showed high levels of activity in frontal cortex. (2) While following a moving object with the eyes, blood flow increased relative to the resting baseline in visual association cortex and in the region of the frontal and supplementary eye fields. (3) While listening to spoken words, activity increased in auditory cortex. (4) While moving the mouth and repeatedly counting to 20, activity increased in the mouth area of motor cortex, supplementary motor area, and auditory cortex. Adapted with permission from Lassen et al. (1978). Copyright © (1978) Scientific American, a division of Nature America, Inc. All rights reserved. ***B***, Averaged event-related responses elicited by sound stimuli in each of 5 participants (1–5) when sound stimuli were fully predictable (certain; solid line) versus when the modality of the same sound stimuli was uncertain (dashed line). In the uncertain condition, sound stimuli occurred on one-third of trials, and visual stimuli occurred on two-thirds of trials. The most dramatic differences occurred in the late positive deflection with a peak latency of ∼300 ms. Positive voltage values are plotted downward. Adapted from Sutton et al., 1965. Reprinted with permission from AAAS.

A mechanistic understanding requires looking inside and measuring activity unfolding in the human brain. Two complementary approaches were pursued in parallel, based on recording brain activity directly using neurophysiology and on imaging metabolic consequences of brain activity through hemodynamic markers. In each case, the scientific roots stretch back to the late 1800s, but the practical methods for investigating the brain mechanisms supporting mental functions noninvasively only started to become available in the 1960s, with the development of event-related potentials (ERPs) enabling cognitive neurophysiology (Walter et al., 1964; Sutton et al., 1965) and of methods to image cerebral blood flow kicking off brain imaging (Lassen et al., 1963). Figure 2 shows some of the earliest examples of each. Thus, the founding of the Society for Neuroscience also coincided with the rise of cognitive neuroscience as we know it today (see also Fig. 1).

Since then, noninvasive human neurophysiology and brain imaging have played major roles in changing and refining our conceptualization of how mind sits in brain. They have moved us away from the earlier view that psychological functions are localized to brain areas that can be combined in series, and instead ushered us toward a network-based organization in which architecture and dynamics play critical roles in prioritizing, routing, and integrating information. Undoubtedly, our current understanding is incomplete, but shaking off the naive, common-sense assumptions and accepting that the fabric of mind may be nonintuitive are an essential first step.

In what follows, we reflect on some of the breakthroughs and insights gained from 50 years of watching the brain at work using noninvasive brain measurements in healthy human volunteers. Inevitably, some relevant tools and developments for studying the human mind fall outside the chosen scope. These include the complementary interference-based stimulation methods for probing causal links between brain activity and behavior (e.g., Barker et al., 1985; Thut and Miniussi, 2009; Herrmann et al., 2013), as well as invasive intracranial human EEG measurements and brain stimulation that can be performed in the context of clinical interventions and monitoring (e.g., Jasper and Penfield, 1949; Lachaux et al., 2003; Chang et al., 2010).

### Brain imaging

Over the last five decades, brain imaging has become the dominant approach for measuring human brain activity. Arguably, its increasing power and granularity to dissect the structural and functional organization of the human brain are major contributors to the success and reach of neuroscience in our society today. The rise and influence of functional brain imaging, especially of the more affordable MRI-based methods introduced in the early 1990s, have been meteoric.

The research impact of brain imaging soon outstripped that of human neurophysiology, which had heretofore been more advanced in its application to understanding human cognition. One interesting question is why imaging gained precedence. An obvious advantage is the intrinsic appeal of an image that maps the loci of activity in the brain compared with the complex waveforms of voltage traces offered by neurophysiology. Another wise move on the part of imagers was co-opting the expertise of experimental psychologists to work alongside those developing the methods to design studies to isolate psychological functions so that their neural correlates could be inferred (e.g., Petersen et al., 1988; Posner et al., 1988). Initial experimental designs used subtraction-based approaches building on mental-chronometry methods (Donders, 1869; Posner, 1978); two conditions equated for all but the critical cognitive operation of interest were compared to estimate the duration, or in the case of brain imaging, the activation, related to that operation. Although it is easy to criticize the approach in hindsight, and to question the validity of the implicit assumption of pure insertion that cognitive operations add linearly, it provided a foothold into the slippery landscape of psychological functions and paved the way for other, more powerful types of designs. But the clinching factor driving the success of imaging studies may be the increasing sophistication of data-analysis methods. Brain-imaging data brought many difficulties that required analytical ingenuity to overcome, such as how to reconstruct images, align them across measurements, account for individual differences in structural and functional anatomy, derive functions to link neural events to their protracted hemodynamic signals, and apportion variability in brain signals to experimental variables. Such challenges attracted significant analytic talent into the field, which resulted in new and ever better ways to interrogate functional brain signals.

The introduction and free dissemination of statistical methods with which to analyze time series of signal fluctuations on a voxel-by-voxel basis as a function of experimental variables (e.g., Friston et al., 1994; Smith et al., 2004) enabled researchers to map out responses of different brain areas to specific psychological functions, but also to capture covariations in activity across brain areas (functional and effective connectivity). Functional measurements taken across brain areas revealed cooperation and influences among areas during a task (e.g., Büchel and Friston, 1997). They also revealed the intrinsic network organization of the brain, with high levels of covariation in areas that are closely functionally related even at rest, when participants have no specific task to perform (Biswal et al., 1995; Raichle et al., 2001; Fox et al., 2005; Damoiseaux et al., 2006). Furthermore, the ability to specify the changing experimental parameters on a trial-by-trial basis provided important mechanistic information about which brain changes impact behavioral performance in a given domain (e.g., Pessoa et al., 2002; Weissman et al., 2006). It also enabled researchers to apply computational modeling to reveal whether and which brain areas are sensitive to latent parameters hypothesized to guide performance (Friston and Dolan, 2010). Multivariate methods building on these foundations were further able to characterize patterns of activations across populations of voxels (e.g., Kamitani and Tong, 2005; Haynes and Rees, 2006), thus indexing the informational content of the processing within an area or network (Kriegeskorte et al., 2006). For example, whereas signals in individual voxels of a typical fMRI study lack the sensitivity to distinguish among different object shapes being viewed, these can be readily decoded from the pattern of activation strengths across sets of voxels (Haxby et al., 2001; Stokes et al., 2009).

### Advances

Detailing all the advances brain imaging has brought to the understanding human cognition would be beyond any reasonable review article. We therefore confine ourselves to highlighting some of the conceptual breakthroughs in our own field of inquiry, selective attention, acknowledging that our choices are biased by our own experience and interests. We will follow a similar approach in subsequent sections highlighting advances based on neurophysiological recording approaches.

PET scanning (Corbetta et al., 1993; Nobre et al., 1997) and later fMRI (Gitelman et al., 1999; Kastner et al., 1999; Corbetta et al., 2000; Hopfinger et al., 2000) vindicated the notion that a large-scale network of brain areas supports the control of visual spatial attention (Mesulam, 1981, 1990). The results played a major role in leaving behind phrenological views and promoting the importance of understanding network-level computations both in regard to attention and more generally in the control of cognition. The network of brain areas involved in controlling attention overlapped significantly with that controlling oculomotor functions (Nobre et al., 1997, 2000; Corbetta et al., 1998), helping highlight the close link between cognitive functions and their sensorimotor building blocks and prompting further investigation of the details of the functional links (e.g., Engbert, 2006; Awh et al., 2006; Ikkai and Curtis, 2011; van Ede et al., 2019a). The increasing spatial resolution of imaging methods combined with retinotopic mapping have helped delineate various parietal and premotor/prefrontal areas that may be relevant to attention control, and promoted further investigations of their causal involvement using transcranial stimulation methods (e.g., Silver et al., 2005; Konen and Kastner, 2008; Szczepanski and Kastner, 2013; Scolari et al., 2015). Studies investigating the consequences of attention modulation showed that many sensory and high-level brain areas were affected by visual-spatial and other forms of attention (Corbetta et al., 1990; Kastner et al., 1998). Rather than locating the site for attention modulation, the findings suggested that attention might affect multiple types and levels of processing. Modulation was also observed in subcortical areas, including thalamic nuclei thought to play early relay functions in visual processing (O'Connor et al., 2002), thus prompting invasive single-unit studies to reassess modulation of thalamic regions by attention (McAlonan et al., 2006, 2008; Wurtz et al., 2011). Imaging studies analyzing functional interactions among brain regions, and subsequent studies combining brain stimulation with imaging, confirmed the top-down influence of parietal and frontal regions on sensory processing (Buchel and Friston, 1997; Moore and Fallah, 2004; Ruff et al., 2006).

MRI studies using multivariate analyses (Haxby et al., 2001; Kamitani and Tong, 2005; Haynes and Rees, 2006; Kriegeskorte et al., 2006) also revealed preactivation of sensory content of task-relevant targets during the anticipatory period (Stokes et al., 2009), in line with findings from single units in monkeys (Chelazzi et al., 1993) and with the influential biased-competition theory of visual attention (Desimone and Duncan, 1995). Interestingly, studies using multivariate approaches further revealed that anticipatory delay activity need not merely reflect a veridical representation of the target template, but instead upregulates the most useful information to guide subsequent performance (Serences et al., 2009), which can consist of systematic distortions of the original template (Scolari and Serences, 2010).

Overall, brain imaging has helped shape our textbook understanding of attention in the human brain (Nobre and Kastner, 2014) and provide a bridge to the studies investigating systems-level and cellular mechanisms in animal models (e.g., Reynolds and Chelazzi, 2004; Reynolds and Heeger, 2009; Fries, 2015; Jonikaitis and Moore, 2019).

### Limitations

The progress made in imaging technology and analysis is undeniable, yet brain imaging on its own is insufficient for understanding the neural basis of adaptive cognition. In some cases, it can even be misleading. Activation maps hide nuances and limitations. They feel immediate and conjure intuitive interpretations, but they are based on indirect markers of neuronal activity. Although the link between brain activity and circulation mediated by metabolic demands is well established (Roy and Sherrington, 1890), the precise linking functions are still being investigated (Raichle and Mintun, 2006). Imaging signals may be systematically biased in ways we are yet to appreciate.

In addition to being indirect, hemodynamic measurements are orders of magnitude slower than their neuronal-activity counterparts. Their timescale, in seconds, is ill suited to investigate psychological processes, many of which unfold over tens to hundreds of milliseconds. The coarse temporal resolution makes it difficult to interpret changes in activation patterns. For example, greater activation in a region could reflect either stronger or lengthier engagement of neuronal populations. More problematically, taking modulations of brain activity in an area at face value can misguide interpretation. If we heed the lesson that neural processes occur within functionally interconnected networks of brain areas, it becomes counterproductive to infer the functional specialization of one individual brain area without high temporal resolution. Through the temporally blurred lens of brain imaging, it can be difficult to distinguish activations resulting from computations within a given area from those initiated in other areas that are fed forward from upstream regions or fed back from downstream regions through network connectivity. Thus, ironically perhaps, brain imaging is most often used to reveal something it cannot, the functional specialization of a given brain area. Imaging studies routinely draw such inferences; but without corroborating evidence from complementary methods with high temporal resolution, it is problematic to attribute the origin of a neural effect to the site of hemodynamic modulation.

For example, in imaging studies, attention-related modulation of visual processing in striate cortex (Kastner et al., 1998; Tootell et al., 1998; Brefczynski and DeYoe, 1999) and lateral geniculate nucleus (O'Connor et al., 2002) could be interpreted as reflecting modulation at very early stages in the feedforward “sweep” of visual processing. However, human neurophysiological studies using similar task designs mostly showed effects of spatial attention starting only later in time, therefore sparing the initial visual potential related to the first feedforward sweep of striate activity (e.g., Clark and Hillyard, 1996; Martínez et al., 1999). These findings suggest that modulation of striate cortex and thalamus in fMRI studies could instead result from reentrant feedback carrying attention-modulated signals from downstream areas (Lamme and Roelfsema, 2000). Resolving such inconsistencies requires careful studies combining both brain imaging and neurophysiological methods in the same task and applying invasive methods with higher combined spatial and temporal resolution.

Studies relying on invasive intracranial recordings in humans illustrate the occurrence of late, reentrant modulation in early sensory regions, which might yield misleading patterns of hemodynamic activations. In a feature-based attention task, participants viewed randomly interleaved red or green words in the center of the screen, and had to follow the narrative of the words in one (attended) color while ignoring words in the other stream (Nobre et al., 1998). Recordings from the posterior fusiform gyrus showed characteristic responses to word stimuli ∼200 ms (Nobre et al., 1994). These were unaffected by the attention manipulation, but strong attention-related modulations occurred much later (after 350 ms), possibly reflecting feedback from different degrees of semantic and contextual integration from attended versus ignored words. Interestingly, in contrast to the intracranial neurophysiological studies showing that early responses in this region are unaffected by lexical or semantic factors (Nobre et al., 1994; Nobre and McCarthy, 1995), fMRI studies have suggested this brain area is sensitive to lexical and contextual semantic factors (e.g., Price and Devlin, 2003; Hauk et al., 2008). An alternative possibility is that imaging-related modulations come from downstream regions as a result of attention or later stages of semantic or contextual analysis.

Intracranial recordings during a contextual-cueing task offer another example of late attention-related modulation in early visual areas (Olson et al., 2001). Identifying a designated target stimulus in a visual-search array is facilitated by repetition of the configuration of distractors (Chun and Jiang, 1998). Implicit long-term memory for the configuration of distractors is proposed to guide spatial attention to the target location to enhance performance (Chun, 2000). Intracranial recordings from visual areas showed late modulation by memory for distractor configurations. The first potentials are unaffected, suggesting modulation as a result of reentrant feedback from later processing stages. Without such qualifications, fMRI findings that activations are modulated in early visual areas (Pollmann and Manginelli, 2010) might be incorrectly interpreted as suggesting that contextual memory influences the earliest stages of visual cortical processing in these tasks.

### Human neurophysiology

Despite its earlier take-off as a method to probe the workings of cognitive functions, noninvasive human neurophysiology later took the backseat position. Recent technical and analytical advances, however, are unleashing the method's full power to investigate information processing and dynamics within human brain networks. Human neurophysiology is once again stepping back into the driver's seat, as researchers increasingly recognize the importance of complementing brain imaging with direct, time-resolved measures of human brain activity to understand brain mechanisms of cognition.

### ERPs

Modern human cognitive neurophysiology took off in the 1960s when methods for averaging EEG traces (Garceau and Davis, 1934; Davis, 1939; Dawson, 1954; Galambos and Sheatz, 1962) made it possible to extract the small yet consistent voltage signals reflecting neural processing systematically related to sensory or cognitive events from the larger ongoing raw voltage fluctuations. Using this method, researchers identified ERPs that were sensitive to cognitive factors during task performance. Pioneering examples were the contingent-negative variation, a large negative voltage buildup following a stimulus that signaled an upcoming behavioral target (Walter et al., 1964), and the late positive component, which varied with the degree of uncertainty about the identity of a target (Sutton et al., 1965) (see Fig. 2*B*).

The ERP method was the first to enable the scientific and noninvasive investigation of cognitive functions in the human brain. The spatial resolution of ERPs is limited by the spatial and temporal summation of voltage signals in the brain and by the ill-posed problem of deriving the intracranial sources from their projection onto the scalp surface (Helmholtz, 1853). Nevertheless, the potentials provide a rich signal of controlled observable variability (Donchin et al., 1978), which can be defined by their latency, amplitude, voltage topography over the scalp, and functional modulation by experimental variables (Allison et al., 1986; Rugg and Coles, 1995). By recording ERPs, it became possible to measure whether and when the human brain was sensitive to a particular type of information or manipulation, and to compare the patterns of responses elicited by different stimuli or under different conditions.

### Advances

The use of ERPs brought many conceptual advances to attention research (Luck et al., 2000; Nobre and Silvert, 2008; Woodman, 2010; Eimer, 2014), and it is noteworthy that ERP reports of attention modulation of sensory processing preceded those using invasive cellular recordings in nonhuman primates (Moran and Desimone, 1985) and noninvasive human brain imaging (Corbetta et al., 1990; Tootell et al., 1995). For example, modulation of sensory potentials during spatial selective-attention tasks (Hillyard et al., 1973; van Voorhis and Hillyard, 1977) broke the impasse on the longstanding debate between “early” and “late” attention, relating to the exact processing stage at which attention operates to prioritize relevant from irrelevant information, either at the level of simple features during perception (early) or only after full stimulus processing through semantic analysis (late). Functional dissociations between modulation of different sensory potentials indicated the existence of multiple modulatory sites (Mangun and Hillyard, 1991; Luck et al., 1994, 1995), negating the stubborn idea of only one bottleneck for information processing and, accordingly, only one site for attention modulation in the human brain (Broadbent, 1958). Studies using word stimuli further showed that modulation could include lexical and semantic content (McCarthy and Nobre, 1993; Bentin et al., 1995), challenging views about the automatic nature of such stages of processing (Deutsch and Deutsch, 1963). ERP studies also clearly showed early sensory modulatory effects of object-based (Valdes-Sosa et al., 1998), feature-based (Hansen and Hillyard, 1983; Leonards et al., 2003; Hopf et al., 2004), and temporal (Doherty et al., 2005) attention, thus helping the field break away from the notion of a privileged type of unit for attention selection (see Nobre and Silvert, 2008; Nobre and Kastner, 2014; Nobre, 2018).

### Limitations

However, the traditional approach to analyzing ERPs dismisses many fundamental sources of variability that are essential for deriving a better, mechanistic understanding of human brain function. Averaging EEG traces into an ERP waveform eliminates trial-by trial variability, thereby greatly reducing the sensitivity to identify the stages of processing that impact behavioral performance. Averaging raw (time-domain) signals also collapses the spectral richness in the EEG signal, recognized from its earliest recordings (Berger, 1929). Frequency-specific patterns in the EEG, whether they reflect truly oscillatory phenomena or shorter-lived signatures of network-related activity (van Ede et al., 2018a), may carry specific functional significance, which still requires direct testing. Finally, averaging also eliminates much of the temporal specificity hailed as the hallmark of neurophysiological methods. Temporal summation of processes triggered by events that overlap in time can make it problematic or impossible to individuate them. Temporal variability in the processes triggered by the same event over multiple trials can also lead to distortions in the overall averaged signal and promote misleading interpretations. For example, a lower-amplitude average potential can result from changes in the strength of a process or its temporal variability across trials.

### Contemporary human neurophysiology

Noninvasive human neurophysiology is experiencing a revival. Interestingly, many of the advances reinvigorating the method have their origin in the very brain-imaging methods that initially overshadowed it in the first place.

The constant quest for improved spatial resolution accentuated by brain imaging also resulted in substantial hardware refinements for noninvasive human neurophysiology. The introduction of MEG has greatly sharpened the spatial resolution of human recording methods (Cohen, 1972; Hari and Salmelin, 1997), and successful efforts are underway to measure magnetic fields generated in the brain with greater flexibility and even greater spatial granularity and signal-to-noise ratio (Boto et al., 2018).

But as with imaging, the clinching factor in improving neurophysiology studies has been the innovation in the concepts and tools used for signal analysis. Many of these are related to, and inspired by, analytical advances introduced for brain-imaging methods. For example, by drawing on advances in imaging, methods for localizing the brain sources of extracranial M/EEG signals in a more distributed, brainwide fashion (e.g., beamforming) (van Veen and Buckley, 1988) have supplemented more traditional dipole-based models (Hämäläinen and Hari, 2002). In tandem, statistical approaches for evaluating the full data space, such as cluster-based permutation approaches (Maris and Oostenveld, 2007), have been adapted and applied to neurophysiological signals. Through such approaches, we have also come to appreciate more fully the richness of neurophysiological data as a key strength of the method. This richness provides a better means to assess “physiological plausibility,” and provides relevant complementary information to *p* values (van Ede and Maris, 2016).

Furthermore, by breaking away from the averaging functions that provided the foundation for initial ERP breakthroughs and embracing the variability in the raw signal, it has become possible to measure signals in their full spectral richness, on a trial-by-trial basis, and to individuate informational content about temporally overlapping events, with high temporal fidelity, and taking temporal variability into account.

### Regaining spectral richness

**Figure 3.**
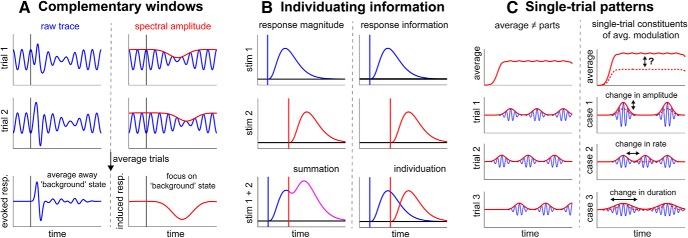
Schematics of innovations in contemporary neurophysiology. ***A***, Raw M/EEG traces (blue) and their spectral amplitudes (red) provide complementary windows into cognitive modulations of neural activity. Spectral analyses enabled researchers to regain “background” states, by enabling the states to be analyzed just like ERP components (i.e., relative to cognitive events and with the increased sensitivity brought by trial averaging). For a relevant example, see Pfurtscheller and Lopes da Silva (1999). ***B***, When multiple stimuli are presented in close temporal proximity, analyses of response magnitudes (ERP and spectral) are complicated by response summation (left column). Decoding analyses that focus on the unique information of the distinct events enable response individuation (right column). For a relevant example, see van Ede et al. (2018b). ***C***, Sustained patterns in trial-average dynamics of, for example, spectral amplitude (as depicted) may reflect the aggregation of many transient burst events at the level of single trials (left column). For a relevant example, see Lundqvist et al. (2016). Accordingly, modulations in average amplitude may reflect a number of distinct changes in the underlying single-trial dynamics (right column). For a relevant example, see Shin et al. (2017).

A key rationale of the ERP approach is that, by repeating the same condition across many trials, we average away the “background” states that obscure the response within the single trials, to reveal the waveform that is common across trials (Fig. 3*A*, left column). The approach assumes that the raw, ongoing activity carries no relevant information-processing content and that it is essentially a nuisance factor to be eliminated.

However, even the earliest EEG recordings clearly suggested the presence of endogenous brain states that are functionally relevant. At rest, the brain was observed to display prevalent patterns of rhythmic activity, such as in the alpha (∼10 Hz) and beta (∼20 Hz) frequencies, which varied systematically with the functional state and neurological condition of the individual (Berger, 1929). Berger's initial observations, and the later realization of resting-state networks using brain imaging (Raichle et al., 2001; Fox et al., 2005; Damoiseaux et al., 2006), suggest that human cognition and behavior are not a reaction to external stimulation, but an interaction between external stimulation and internal brain states. Capturing and characterizing these internal states, and understanding their relation to the processing of external inputs, could therefore be fundamental for understanding psychological functions.

From the beginning, these rhythms were shown to vary with mental acts. For example, in addition to the strong effects of closing versus opening the eyes on inducing and suppressing the alpha rhythm, engaging in an attention-consuming act, like performing a difficult mental arithmetic calculation, also strongly diminished the alpha rhythm (Berger, 1929; Adrian and Matthews, 1934). Thus, the ERP method dismissed as “noise” internal functional brain states carrying characteristic spectral signatures. It is now widely recognized that such brain states can provide complementary windows into neural and cognitive computations (e.g., Hari and Salmelin, 1997; Klimesch, 1999; Pfurtscheller and Lopes da Silva, 1999; Siegel et al., 2012).

The reclaiming of the spectral dimension took off with the application of time-resolved spectral analyses that allowed researchers to analyze spectral modulations in an ERP-like fashion (Fig. 3*A*, right column) (Pfurtscheller and Aranibar, 1979; Pfurtscheller and Lopes da Silva, 1999) and construct comprehensive time-frequency plots (e.g., Tallon-Baudry and Bertrand, 1999). Complementing these developments, methods have been developed to derive the spectral characteristics of brain activity empirically (Huang et al., 1998, 2016). These methods sidestep some of the problematic consequences that occur when brain signals violate assumptions that are inherent in conventional Fourier-based methods.

In human attention research, a prominent example of the utility of spectral analyses comes from studies linking ongoing alpha oscillations to selective attention and perception. Rather than dismissing alpha oscillations as a background state, Foxe et al. (1998) and Worden et al. (2000) demonstrated that anticipatory states of attention are associated with a relative attenuation of alpha oscillations in brain areas that code for the anticipated stimulus. We and others have subsequently shown that such alpha attenuation also tunes in to relevant moments in time (Rohenkohl and Nobre, 2011; van Ede et al., 2011; Zanto et al., 2011; Heideman et al., 2018) and that such states enhance sensory processing (e.g., Hanslmayr et al., 2007; van Dijk et al., 2008; Romei et al., 2010; Gould et al., 2011; van Ede et al., 2018b) and upregulate firing rates in the underlying populations (Haegens et al., 2011). On these bases, relative alpha attenuation in task-relevant sensory brain areas has been proposed to serve a key “gating mechanism” of the human brain (e.g., Jensen and Mazaheri, 2010; Foxe and Snyder, 2011), including for gating perceptual representations within working memory (Wallis et al., 2015; van Ede, 2018).

The analyses of spectral states have also helped extract and characterize “resting-state” networks from human neurophysiological recordings, by quantifying common amplitude fluctuations across brain regions (Mantini et al., 2007; Brookes et al., 2011; Hipp et al., 2012). This has enabled a bridge to canonical networks observed in imaging (Raichle et al., 2001; Fox et al., 2005; Damoiseaux et al., 2006) while also enabling the study of the dynamics of these networks at cognitively relevant time scales (de Pasquale et al., 2010; Baker et al., 2014; Florin and Baillet, 2015; Vidaurre et al., 2018). Though it is still early days, this has already yielded new perspectives on the transient nature of network activations, which inevitably remains hidden in imaging methods, and on the role of such network dynamics in perception and attention (e.g., Weisz et al., 2014; Astle et al., 2015).

These examples showcase how the incorporation of the spectral dimension is having a major impact on the field. It has afforded a new complementary dimension within the same signal we have always collected. The rich nature of this dimension makes it possible to characterize neural states in time and frequency, and to bridge physiological and imaging studies of human brain activity.

### Tracking informational content

Machine learning has also found its way to human neurophysiology. Building on earlier applications of multivariate decoding analyses in brain imaging (Haxby et al., 2001; Kamitani and Tong, 2005; Haynes and Rees, 2006; Kriegeskorte et al., 2006), decoding is becoming mainstream in neurophysiological studies of human cognition (e.g., King and Dehaene, 2014; Cichy et al., 2015; Stokes et al., 2015). Unlike conventional analyses of ERPs and spectral modulations that capture response magnitudes, which may relate to changes in processing in many ways, decoding analyses can capture the content-specific information contained in the signal, providing more direct insights into the quality of representation (Kriegeskorte et al., 2006). Using such analyses with human neurophysiology allows the tracking of informational content through time and reveals the dynamic nature of neural coding (Stokes et al., 2013; King and Dehaene, 2014). Initial studies showed that information content in M/EEG measurements may be present earlier than in conventional ERP/Fs (Ramkumar et al., 2013), and a wave of recent studies has started to shed new light on attentional dynamics in perception and memory (e.g., LaRocque et al., 2013; Garcia et al., 2013; Marti et al., 2015; Myers et al., 2015; Foster et al., 2017; Wolff et al., 2017; van Ede et al., 2019b). For example, while it has long been known that anticipation amplifies early visual responses (e.g., the visual P1 and N1 ERP components) (Mangun and Hillyard, 1987, 1991; Luck et al., 1994), up until recently, it had remained unclear whether anticipation also enhances the quality of information linked to stimulus identity within these early brain responses. We recently demonstrated precisely this (van Ede et al., 2018b). Interestingly, the attention-related gain in information was uncorrelated with the amplification of the visual potential in the same time window, suggesting that response amplitude and response information provide complementary windows into attentional operations (for an example of a similar notion in fMRI, see also Kok et al., 2012).

### Individuating representations

Decoding analyses also provide a powerful tool to overcome another limitation of conventional analyses of response amplitudes. When multiple items occur together or in rapid succession, the amplitude of the neural response will reflect the aggregate response to the sum of the stimuli (Fig. 3*B*, left column, bottom). From this summed response, it is notoriously hard to partition the response into the components associated with the individual stimuli. This becomes even more problematic when deciphering the origin of a cognitive modulation that rides on the aggregate response.

Decoding analyses provide ways around this because they individuate items based on their unique representational information, thereby enabling the tracking of multiple representations in time concurrently (Fig. 3*B*, right column, bottom). We recently used this approach to study how anticipation facilitates the processing of visual targets when these compete with temporally adjacent visual distractors (van Ede et al., 2018b). By decoding stimulus-identity information from the EEG, we were able to individuate both target and distractor codes despite their temporal proximity, and to track the sensory processing of each over time. This revealed that anticipation enhances the quality of the target representation and delays interference from the distractor on the target processing, providing a protective temporal window for high-fidelity target processing. Similar approaches have started to reveal new insights into other dynamic phenomena, such as the attentional blink (Marti and Dehaene, 2017), the matching of mnemonic templates to visual inputs (Myers et al., 2015), multitasking (Marti et al., 2015), and the concerted selection of visual and motor representations from working memory (van Ede et al., 2019b).

Complementing decoding analyses that capitalize on informational content, individuation can also be achieved through frequency tagging (e.g., Brown and Norcia, 1997; Tononi et al., 1998), an approach that dates back to early days of cognitive neurophysiology (Lansing, 1964), while still subject to contemporary developments (Zhigalov et al., 2019). Here, items are presented (tagged) at distinct, separable, frequencies. By titrating the analysis according to the tagged frequencies, it becomes possible to isolate the neural response to the distinct items, and to track the amplitudes of these item-specific responses over time. Such an approach has been used, for example, to track the concurrent focusing of attention at spatially segregated locations (Müller and Hübner, 2002; Müller et al., 2003) or to track the neural dynamics of feature-based attention on spatially overlapping stimuli (Baldauf and Desimone, 2014).

### Regaining single-trial dynamics

Recent years have also seen an increased emphasis on the importance of single-trial dynamics. While combining data yields clean and robust signals, it risks two potential fallacies: (1) assuming trialwise variability is noise and (2) treating the average as a prototypical reflection of the underlying dynamics.

Regarding trialwise variability, we have learned that, even within a single experimental condition, neural variability can predict variability in task performance (e.g., van Dijk et al., 2008; Mazaheri et al., 2009; Jones et al., 2010; van Ede et al., 2012; Cravo et al., 2013; Myers et al., 2014). Rather than noise, such fluctuations may reflect spontaneous fluctuations in cognitive state (see also Nienborg and Cumming, 2009). Capturing this variability can give relevant insights into the relation between brain states and cognition, complementing insights derived from analyzing systematic differences across experimental conditions. In addition to variability in the strength of responses, variability may also occur in the temporal cascade of processing. Such variability can smear and dampen ERP amplitudes and may lead to “false dynamics” in cross-temporal decoding analyses (King and Dehaene, 2014) that assume consistent timing of neural processing across trials (Vidaurre et al., 2019). New temporally unconstrained decoding models are on the rise to capture this temporal variability, and these models too can account for variability in behavioral performance (Vidaurre et al., 2019). Such approaches promise to reveal important principles about the time course of neural computations relevant to cognition, helping to arbitrate between proposals suggesting successive stable states of neural processing (e.g., Pascual-Marqui et al., 1995; Khanna et al., 2015) versus a continuous unfolding of dynamic neural activity best captured as a trajectory through state space (e.g., Buonomano and Maass, 2009).

There is also increased appreciation that the trial-averaged response may provide a highly misleading model of the underlying response, or the underlying patterns of activity. For example, the classic “ramp-like” activity in the trial average may reflect the averaging of many “step-like” patterns with jittered timings across trials (Latimer et al., 2015; Stokes and Spaak, 2016). Likewise, sustained patterns in average time-frequency maps of spectral amplitude may reflect the aggregation of many short-lived, isolated “burst events” at the single-trial level, which happen to ring in a particular frequency band (Feingold et al., 2015; Lundqvist et al., 2016; Shin et al., 2017) (Fig. 3*C*, left column).

While the physiological interpretation of such putative burst events is still open to debate (van Ede et al., 2018a), their possibility has prompted researchers to dissect how cognitive factors affect their putative constituent parameters at a single-trial level, such as changes in amplitude, rate, timing, and duration of burst events (Fig. 3*C*, right column). In principle, the increased granularity and quantification of these event parameters may provide closer correspondence with the underlying physiology and help chart links between neurophysiological events and cognition. In attention research, for example, attenuation of beta activity in anticipation of a tactile target (as in Jones et al., 2010; van Ede et al., 2011, 2012) has been linked specifically to a decrease in the rate of punctate beta bursts, which also predicts perceptual performance (Shin et al., 2017).

### Future outlook

Over the last 50 years, we have learned a lot about what is happening under the mind's hood. The tools are improving all the time. Yet, we are far from done understanding this mysterious motor. If our explorations have taught us one lesson, it is to remain openminded. Sometimes we tend to place too much value in our local interpretations and be overly dismissive of alternative or additional possibilities. This slows progress. In the context of this review, we have highlighted some past examples, such as the phrenological importance of specific brain areas, the primacy of brain responses triggered by external events, the disregard for spectral signals, and the disregard for variability of the strength and timing of neural events. We may think we are smarter now, but most likely we are blind to some current traps. For example, are the canonical resting-state networks of today too rigid of a concept, akin to a new version of the old phrenological unit and similarly prone to mask the true level of flexibility and interaction in the brain? Who knows, but why put on blinkers when the adventure is getting so interesting?

In building the future toolkit, we must remember that mind and brain make behavior. To investigate the link, we need to start applying the same level of ingenuity to develop the means with which to investigate the complexity and richness of behavior as we have applied to understanding the brain. Incredibly, typical studies of human cognition involve behavioral measures confined to simple individual responses, such as the accuracy and timing of a button press or of an eye movement. It is time that we upgrade to measures of trajectories, force, hesitations, postural relations, activity across muscles; and that we let our participants stand up, move around, and interact in real or virtual environments. Methods for capturing various aspects of immersive behavior are being developed, often within the contexts of the tech and entertainment industry, simulation training, or clinical rehabilitation. Neuroscientists interested in human cognition and behavior need to step up their game and contribute to the refinement of these methods as well. Doing so will also prompt innovations in how we measure brain activity. Exciting developments in methods for measuring brain activity in natural environments and during normal active behavior are afoot (e.g., De Vos et al., 2014; Boto et al., 2018).

In a similar spirit, we must not treat the brain as an isolated organ, but remember it is part of a much larger ecosystem: the body. As a consequence, many signals in the periphery provide complementary windows into the neural basis of cognitive processes, even when these processes are conventionally considered to be “covert” (e.g., Hafed and Clark, 2002; Engbert and Kliegl, 2003; van Ede and Maris, 2013; Corneil and Munoz, 2014). Two striking examples of this come from our own recent work on attentional operations in working memory, revealing microsaccadic gaze biases (van Ede et al., 2019a) and pupil dilations (Zokaei et al., 2019) during purely internal attentional focusing. Nor should we forget the reverse direction of influence: many inputs to the brain come from other organs of the body, and these inputs too may interact with neural processes linked to cognition (e.g., Park et al., 2014; Azzalini et al., 2019).

The final frontier will be to forge a much closer relationship between human cognitive neuroscience and the rest of neuroscience research. No matter how far along noninvasive methods for watching the brain at work have come, complementary approaches are required for testing the causal contribution of activity in brain networks to human cognition, such as interference-based stimulation methods or neuropsychological testing of individuals with lesions or damage to brain areas or networks. In addition, a deep understanding of the human mind, brain, and behavior will require integration with findings from methods at the systems, cellular, and molecular levels, which provide finer spatial and temporal resolution for measuring signals as well as for manipulating or interfering with brain signals. Integrating across levels of organization is much more difficult than working with any one level, but this should not deter us. After all, it is where it all comes together.
